# Activation of peroxymonosulphate using a highly efficient and stable ZnFe_2_O_4_ catalyst for tetracycline degradation

**DOI:** 10.1038/s41598-023-38958-1

**Published:** 2023-08-25

**Authors:** Xuying Zhao, Wei Li, Junyi Gao, Caibin Li, Yansong Xiao, Xue Liu, Dean Song, Jiguang Zhang

**Affiliations:** 1 Institute of Tobacco Research, Chinese Academy of Agricultural Sciences, Qingdao, 266101 China; 2 China Tobacco Jiangsu Industrial Co., Ltd., Nanjing, 210019 China; 3 Bijie Tobacco Branch Company of Guizhou Province, Bijie, 551700 China; 4 Chenzhou Tobacco Company of Hunan Province, Chenzhou, 423000 China

**Keywords:** Chemistry, Catalysis

## Abstract

Tetracycline (TC) is a widely used antibiotic that adversely affects ecosystems and, therefore, must be removed from the environment. Owing to their strong ability to oxidise pollutants, including antibiotics, and selectivity for these pollutants, an improved oxidation method based on sulphate radicals (SO4·^−^) has gained considerable interest. In this study, a novel technique for removing TC was developed by activating peroxymonosulphate (PMS) using a ZnFe_2_O_4_ catalyst. Using the co-precipitation method, a ZnFe_2_O_4_ catalyst was prepared by doping zinc into iron-based materials, which increased the redox cycle, while PMS was active and facilitated the production of free radicals. According to electron paramagnetic resonance spectroscopy results, a ZnFe_2_O_4_ catalyst may activate PMS and generate SO_4_·^−^, HO·, O_2_·^−^, and ^1^O_2_ to eliminate TC. This research offers a new method for creating highly effective heterogeneous catalysts that can activate PMS and destroy antibiotics. The study proposes the following degradation pathways: hydroxylation and ring-opening of TC based on the products identified using ultra-performance liquid chromatography-mass spectrometry. These results illustrated that the prepared ZnFe_2_O_4_ catalyst effectively removed TC and exhibited excellent catalytic performance.

## Introduction

Recently, due to the rapid development of industry, more and more antibiotic pollutants are discharged into the water body, resulting in increasingly serious water pollution. Tetracycline (TC) is a widely used antibiotic in medical and livestock farming^1^. However, a significant amount of TC is released into the environment and is not absorbed by humans or animals, resulting in increased microbial resistance and harmful impacts on ecological system^2,3^. TC levels as high as 20 mg/L have been reported in aquaculture wastewater, and recently, TC has been detected in drinking water^4,5^. Therefore, effective methods for the removal of TC from aqueous solutions have become a matter of urgent concern.

In recent years, antibiotics have been removed from water using various methods such as adsorption, biodegradation^6^, photodegradation^7,8^, and advanced oxidation processes (AOPs)^9^. The conventional methods such as adsorption and membrane processes often have some limitations including production of secondary pollutants, high cost and tedious process. The use of AOPs, in which large organic molecules are converted into small organic molecule compounds and even H_2_O and CO_2_, is the most effective method for removing TC^10,11^.Advanced oxidation process based on peroxymonosulfate (PMS) activation emerged as one of the most promising technologies for antibiotics remediation.

SO_4_·^−^ has a higher oxidation potential, longer duration, and wider pH range compared to HO· produced via the Fenton reaction^12^. Generally, persulfates such as PMS or peroxydisulfate (PDS) are used to produce SO_4_·^–^^13–15^. PMS with an asymmetric structure exhibits a stronger oxidation performance than PDS with a solid symmetric framework^16^. Therefore, PMS is widely used in radical sulphate-based AOPs, which are typically facilitated by using metal-containing catalysts (Co, Fe, Cu, and Mn)^17–22^. However, highly effective, reliable, and recyclable heterogeneous catalysts for practical applications are still required. Heterogeneous catalyst is preferred as PMS activator compared to homogeneous catalyst due to the ease of catalyst recovery, minimum release of secondary pollutant and the potential to operate under such extreme conditions (high pressure and high temperature).

Among the transition-metal-based catalysts, Fe-based catalysts are frequently utilised to trigger PMS because of their high effectiveness, safety, non-toxicity, and low price. Fe-based catalysts that can activate PMS to breakdown organic pollutants include magnetic Fe_3_O_4_, α-Fe_2_O_3_, γ-Fe_2_O_3_, and δ-FeOOH^23^. Although low valence Fe^2+^ and Fe^0^ are readily oxidised, the slow cycle of Fe^2+^/Fe^3+^ results in low PMS activation efficiency^24^. Therefore, to eliminate these negative effects, bimetallic oxide catalysts have been proposed as substitutes for improving catalytic activity and stability. In the past, there were many studies on the degradation of organic pollutants by persulfate using Fe–Mn, Fe–C_O_ bimetallic oxides as catalysts^20,22,24^, but the leaching of C_O_ and Mn ions will cause secondary pollution to the environment, therefore, this study focused on environmental pollution-free Fe–Zn bimetallic oxide catalysts.

In this study, ZnFe_2_O_4_ catalyst was synthesised using the co-precipitation reaction, and its performance in TC degradation was evaluated. The primary goals of this study were to (1) investigate the physicochemical characteristics of the catalyst and discuss its catalytic effectiveness in PMS systems, (2) investigate the effect of various environmental conditions on TC degradation, and (3) examine the reactive oxygen species generated in the ZnFe_2_O_4_/PMS system and elucidate the TC degradation process. Our study provides a new perspective on finding improved, inexpensive, and eco-friendly catalysts.

## Methods

### Materials

PMS (KHSO_5_·0.5KHSO_4_·0.5K_2_SO_4_), iron hexahydrate chloride (FeCl_3_·6H_2_O), zinc chloride (ZnCl_2_), TC, sodium chloride (NaCl), sodium carbonate (Na_2_CO_3_), and sodium dihydrogen phosphate (NaH_2_PO_4_) were supplied by Aladdin Industrial Co., Ltd. Hydrochloric acid (HCl), ethanol, and sodium hydroxide (NaOH) were purchased from Guangzhou Chemical Reagent Co. Deionied water (DI) was used throughout the experiment. All reagents were of the lowest analytical quality.

### Synthesis of ZnFe_2_O_4_

Using a co-precipitation method, ZnFe_2_O_4_ was synthesised. First, FeCl_3_·6H_2_O and ZnCl_2_ were ultrasonically dispersed in 50 mL of DI water for 30 min. The resultant mixture was then placed in a water bath at 50 °C and stirred magnetically. NaOH was added to the solution until the pH reached 9, and the suspension was continuously stirred and maintained at 50 °C for 1 h. The ZnFe_2_O_4_ composites were then centrifuged, separated, and washed with ethanol and ultrapure water to reach a pH of 7. The obtained composites were dried at 60 °C for 24 h, pulverised, passed through an 80-mesh sieve, and then calcined at 600 °C for 2 h under N_2_ gas.

### Characterization of the catalyst

The X-ray diffraction (XRD) pattern were measured between 5 and 80° at 40 kV and 250 mA. The morphology of the ZnFe_2_O_4_ composites was obtained using scanning electron microscopy (SEM; TESCAN MIRA LMS) equipped with energy-dispersive X-ray spectroscopy (EDS). X-ray photoelectron spectroscopy (XPS) was performed using a Thermo Scientific K-Alpha photoelectron spectrometer system.

### Catalytic activity test

To evaluate the catalytic performance and reusability of ZnFe_2_O_4_, we determined its TC removal efficiency. To achieve an equilibrium between adsorption and desorption, the catalyst (0.2 g/L) was uniformly suspended in the TC solution (20 mg/L) at 25 °C and continuously shaken for 30 min. To initiate the catalytic oxidation, PMS was introduced into the reaction solution. We collected 2 mL of sample solutions at a time, filtered them through a 0.22 mm membrane, and measured their TC content using an ultraviolet–visible spectrophotometer at 360 nm. An ultra-performance liquid chromatography-mass spectrometry (UPLC-MS) system was used to identify the TC oxidation intermediates. Reactive oxygen species (ROS) were detected using electron paramagnetic resonance (EPR). After the reaction, the catalyst was collected, washed with ethanol and water, and used in a subsequent cycle to determine its suitability for reuse.

## Results and discussion

### Characterization of catalysts

**Figure 1:**
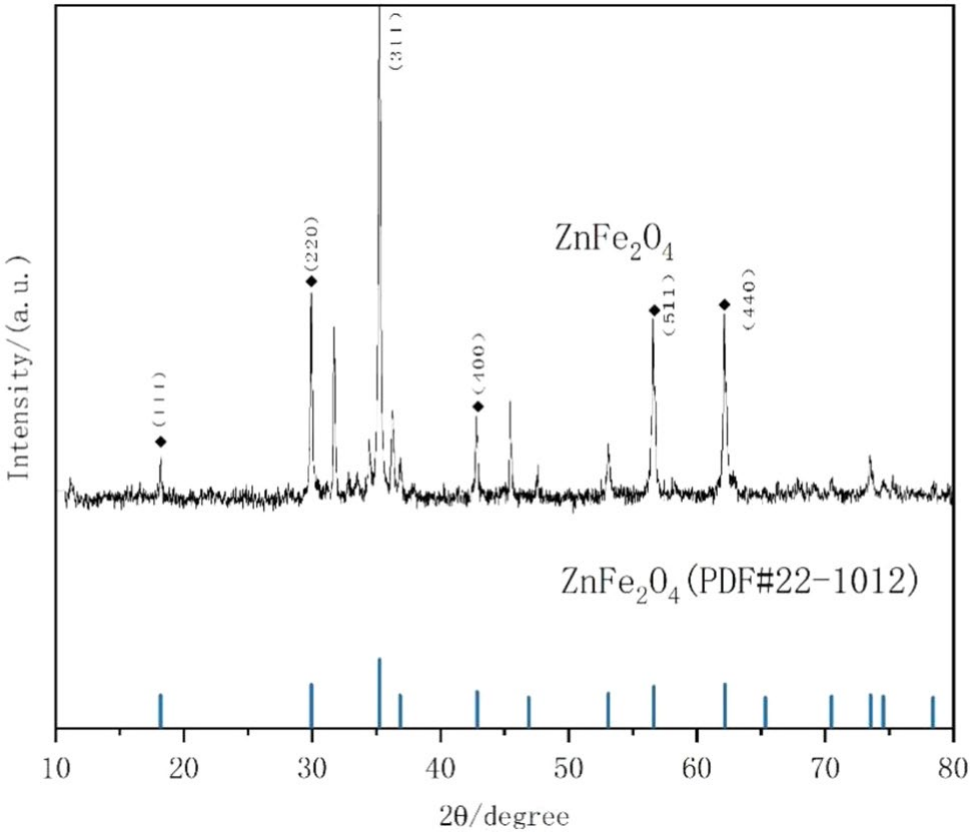
X-ray diffraction pattern of the synthesized ZnFe_2_O_4_ catalyst.

The XRD pattern of the prepared catalyst matched that of cubic spinel ZnFe_2_O_4_ (JCPDS file No. 22-1012), indicating the presence of ZnFe_2_O_4_ crystals (Fig. 1). Six diffraction peaks were observed at 2*θ* = 18.19°, 29.92°, 35.26°, 42.84°, 45.45°, and 62.21°, which corresponded to the (111), (220), (311), (400), (511), and (440) planes of the ZnFe_2_O_4_, respectively, indicating its high surface purity and good crystallinity.

**Figure 2:**
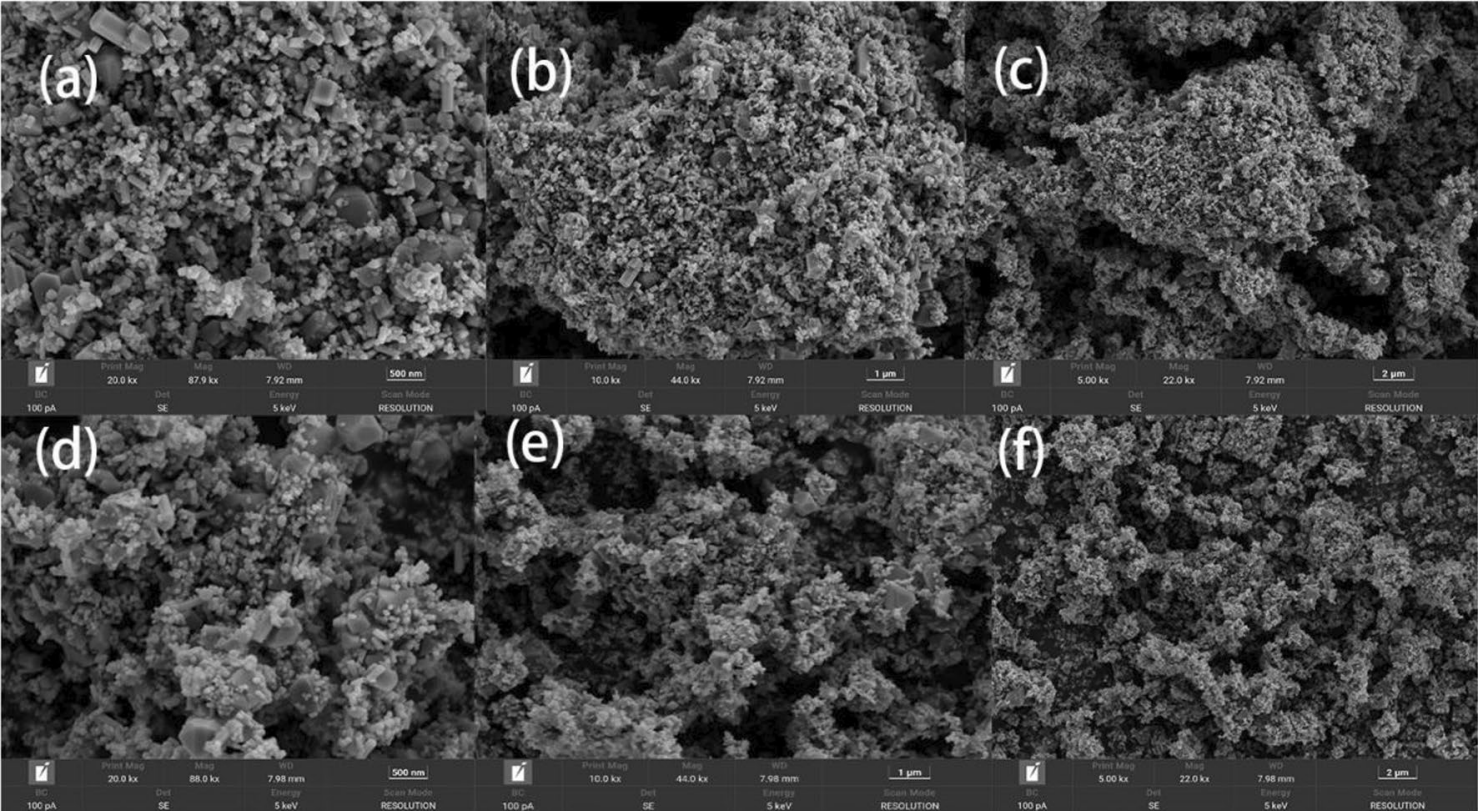
SEM images of ZnFe_2_O_4_ particles before and after the reaction. Higher magnification suggests the presence of agglomerated nanoparticles.

The SEM was used to examine the surface morphology and particle size distribution of ZnFe_2_O_4_. Figure 2 shows an SEM image of a synthetic ZnFe_2_O_4_ catalyst at various magnifications. Evidently, ZnFe_2_O_4_ nanoparticles with hexagonal and spherical structures are uniformly dispersed. After the reaction, the surface pores of the catalyst become larger.The homogenous distribution of the ZnFe_2_O_4_ particles may help in establishing contact between the catalyst and oxidant, facilitating the activation of PMS^25^. Moreover, the surface of ZnFe_2_O_4_ contains several pores, which help adsorb TC on the catalyst surface.

### TC degradation in various systems

**Figure 3:**
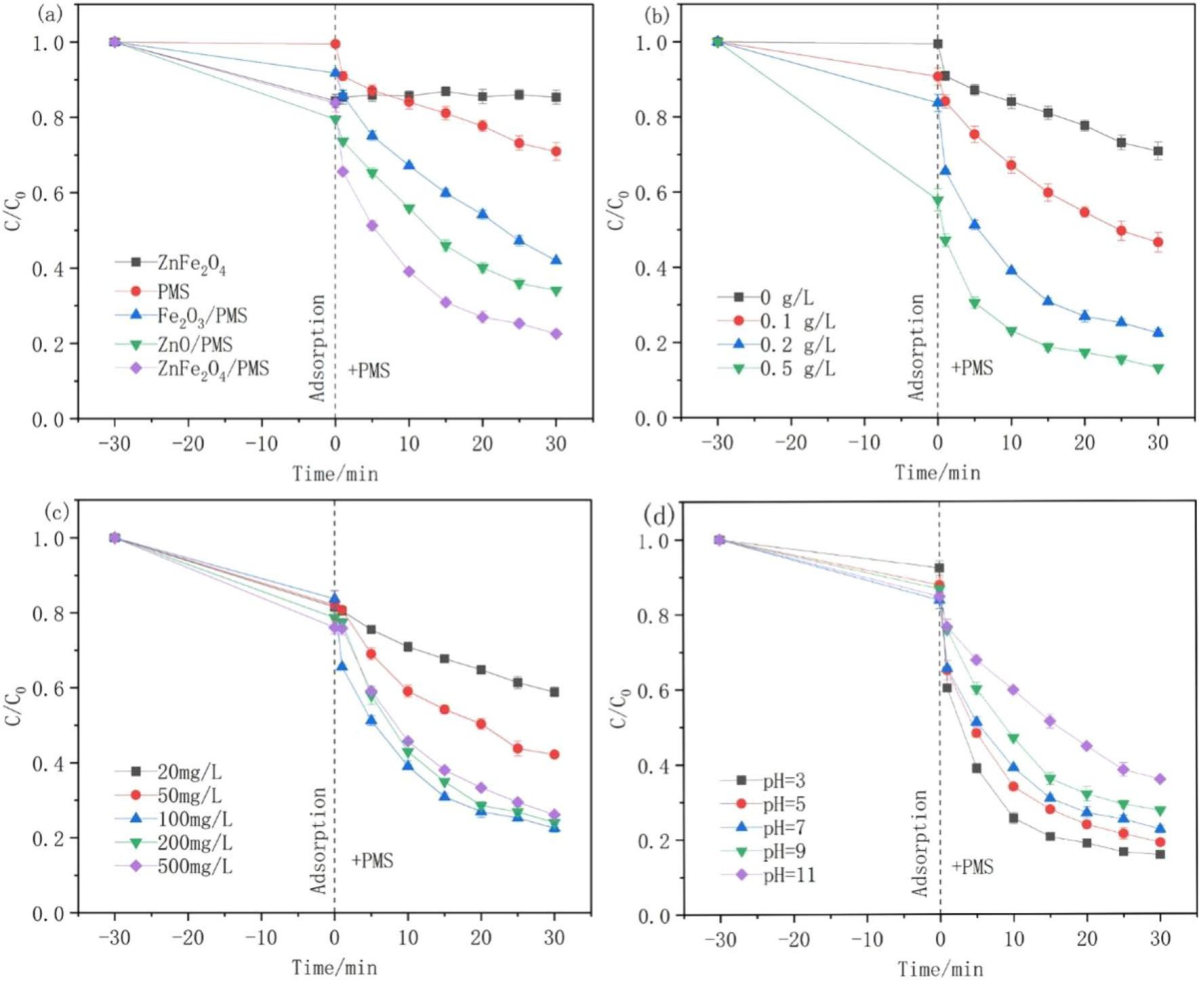
Effects of (**a**) different catalysts, (**b**) catalyst dosage, (**c**) PMS dosage, (**d**) initial pH on TC degradation (Unless stated, [Catalyst] = 200 mg L^−1^, [TC] = 20 mg L^−1^, [PMS] = 100 mg L^−1^, pH = 7).

The degradation of tetracycline under different Fe-based catalysts was illustrated in Table 1. TC removal efficiency was higher (63%) than other Fe-based catalysts, when Fe–Zn catalysts was employed in the same reaction conditions. So, the TC removal efficiencies were investigated in various systems to evaluate the catalytic effectiveness of ZnFe_2_O_4_-activated PMS for TC degradation (Fig. 3a). ZnFe_2_O_4_ alone was found to remove 15% of TC within 30 min because of TC adhering to the large surface area of the catalyst. Despite being a powerful oxidant (E = 1.82 V), PMS alone could remove only 30% of the TC in a 60-min period due to insufficient catalyst for PMS activation and oxygen radical production. The TC removal efficiencies of Fe_2_O_3_ and ZnO as catalysts were 60% and 66%, respectively, demonstrating that both ZnO and Fe_2_O_3_ can activate PMS. However, the TC degradation efficiency could reach 78% in ZnFe_2_O_4_/PMS system, indicating its improved catalytic performance.

**Table 1:** Comparison of performance of different Fe-based catalysts.

Catalyst (Dose, g/L)	PMS (g/L)	Pollutants	Degradation efficiency (%)	Reaction time (min)
Fe–Sn (0.1)	0.1	TC (20 mg/L)	56	60
Fe–Cu (0.1)	0.1	TC (20 mg/L)	44	60
Fe–Al (0.1)	0.1	TC (20 mg/L)	58	60
Fe–Zn (0.1)	0.1	TC (20 mg/L)	63	60

### Catalytic activity and reaction parameter effects

The effects of the catalyst amount, PMS addition amount, and starting pH on TC decomposition were investigated to determine the catalytic oxidation efficiency of ZnFe_2_O_4_. The TC degradability improved as the catalyst quantity was increased from 0.1 to 0.5 g L^−1^, as shown in Fig. 3b. With a catalyst concentration of 0.1 g L^−1^, 53% TC was removed in 60 min. This is attributed to the increased number of active sites available for PMS activation as the catalyst dose increases to facilitate TC degradation^26^. When the catalyst concentration was increased to 0.2 g L^−1^, TC removal increased to 78% in 60 min. However, no discernible improvement was observed on further increasing the catalyst amount to 0.5 g L^−1^, which may be attributed to the ability of the catalyst to bind excess radicals that tend to aggregate^27–30^. Figure 3c shows the effect of the PMS concentration on the TC removal efficiency. Using 20 mg L^−1^ PMS, the TC elimination was 42% within 60 min and reached to 78% when using 100 mg L^−1^ PMS. This could be because increasing the PMS concentration increases the contact between PMS and the catalyst, thus producing more free radicals^31^. Nevertheless, the effectiveness of TC elimination decreased when the PMS concentration was increased further. Because the excess PMS could quench SO_4_·^−^ and HO· to form SO_5_·^−^ (Eqs. 1 and 2) with weak oxidation ability^32^, the produced SO_4_·^−^ or SO_5_·^−^ should also have a self-quenching ability (Eqs. 3 and 4)^33–36^. Therefore, in future studies, 100 mg/L PMS should be used as the optimal concentration.

1 $$ {\text{HSO}}_{{5}}^{ - } + {\text{ SO}}_{{4}} \cdot^{ - } \to {\text{ SO}}_{{5}} \cdot^{ - } + {\text{ H}}^{ + } + {\text{ SO}}_{{4}}^{{{2} - }} $$

2 $$ {\text{HSO}}_{{5}}^{ - } + {\text{ HO}} \cdot \, \to {\text{ SO}}_{{5}} \cdot^{ - } + {\text{ H}}_{{2}} {\text{O}} $$

3 $$ {\text{SO}}_{{4}} \cdot^{ - } + {\text{ SO}}_{{4}} \cdot^{ - } \to {\text{ S}}_{{2}} {\text{O}}_{{8}}^{{{2} - }} $$

4 $$ {\text{SO}}_{{5}} \cdot^{ - } + {\text{ SO}}_{{5}} \cdot^{ - } \to {\text{ S}}_{{2}} {\text{O}}_{{8}}^{{{2} - }} + {\text{ O}}_{{2}} $$

A key factor influencing TC removal is the initial pH of the reaction solution. Figure 3d shows that the TC removal efficiencies within 60 min were 85%, 81%, 78%, 73%, and 64% at different pH values of 3.0, 5.0, 7.0, 9.0, and 11.0, respectively. According to these findings, ZnFe_2_O_4_-activated PMS could dissolve PMS over a broad pH range, and its removal effectiveness decreased with increasing pH. Generally, SO4·^−^, which can be manufactured in large quantities for TC degradation, is the primary active species under acidic conditions (Eqs. 5 and 6)^37,38^. However, SO_4_·^−^ tends to react with OH^−^ to form HO· under alkaline conditions (Eq. 7)^39^. To dissociate TC chemical bonds, the oxidative potential of the HO· radical should be smaller than that of the SO_4_·^−^ radical. A higher concentration of OH^−^ can also cause HO· to interact with OH^−^, resulting in radical annihilation and reduced TC degradation efficiency.

5 $$ {\text{S}}_{{2}} {\text{O}}_{{8}}^{{{2} - }} + {\text{ H}}^{ + } \to {\text{ HS}}_{{2}} {\text{O}}_{{8}}^{ - } $$

6 $$ {\text{HS}}_{{2}} {\text{O}}_{{8}}^{ - } \to {\text{ SO}}_{{4}} \cdot^{ - } + {\text{ SO}}_{{4}} \cdot^{ - } + {\text{ H}}^{ + } $$

7 $$ {\text{SO}}_{{4}} \cdot^{ - } + {\text{ OH}}^{ - } \to {\text{ SO}}_{{4}}^{{{2} - }} + {\text{ HO}} \cdot $$

### Effects of different anions on TC degradation

**Figure 4:**
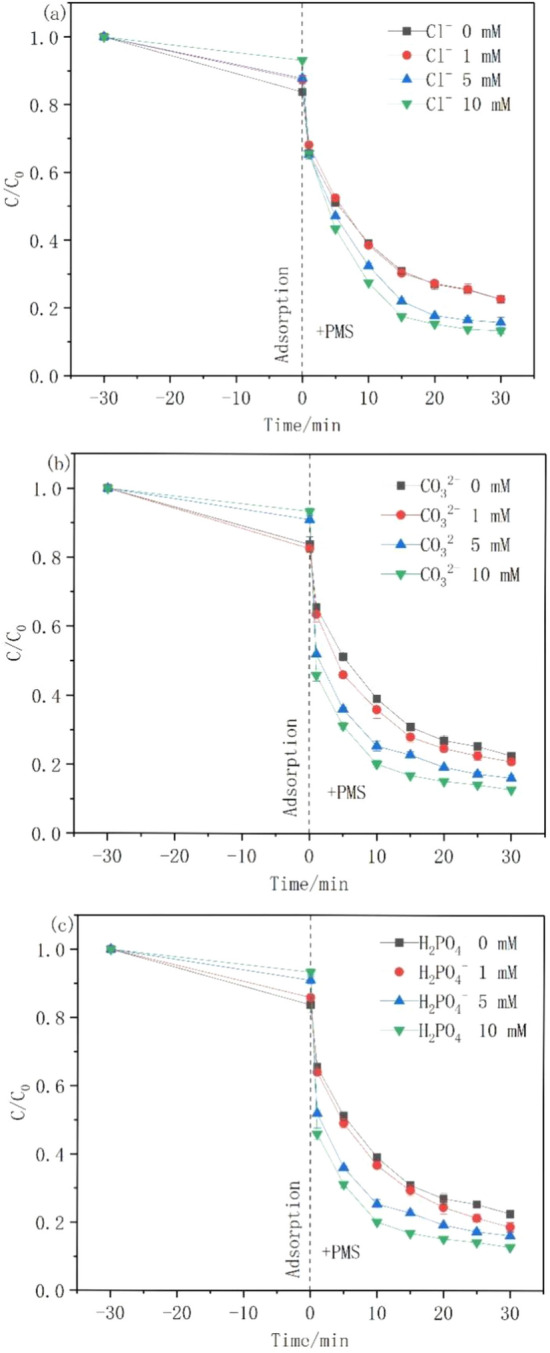
Effects of co-existing (**a**) chloride, (**b**) carbonate, and (**c**) phosphate ions on TC degradation ([Catalyst] = 200 mg L^−1^, [TC] = 20 mg L^−1^, [PMS] = 100 mg L^−1^, pH = 7).

Water and wastewater contain various inorganic anions that affect tetracycline removal. Therefore, the effects of Cl^−^, ${\text{CO}}_{3}^{2}$, and ${\text{H}}_{{2}} {\text{PO}}_{{4}}^{ - }$ on the rate of TC degradation in the ZnFe_2_O_4_/PMS system were studied. Figure 4a shows that 1 mM of Cl^−^ had a slight effect on TC degradation; however, 5–10 mM of Cl^−^ could improve TC removal efficiency to 87%. Furthermore, high levels of Cl^−^ may transfer electrons to PMS, resulting in sulphate radicals and superabundant chlorine species (Eqs. 8 and 9)^40–43^, which may participate in the TC degradation process^44^.

8 $$ {\text{Cl}}^{ - } + {\text{ ROS }} \to {\text{ Cl}} \cdot $$

9 $$ {\text{Cl}} \cdot \, + {\text{ HSO}}_{{5}}^{ - } \to {\text{ SO}}_{{4}} \cdot^{ - } + {\text{ HOCl}} $$

Moreover, increasing the ${\text{CO}}_{{3}}^{{{2}{-}}}$ concentration from 1 to 10 mM facilitated TC breakdown (Fig. 4b). This might be attributed to the activation of asymmetrically structured PMS by ${\text{CO}}_{{3}}^{{{2}{-}}}$, thus producing more reactive free radicals (Eq. 10)^45–47^.

10 $$ {\text{CO}}_{{3}}^{{{2} - }} + {\text{HSO}}_{{5}}^{ - } + {\text{H}}^{ + } \to {\text{SO}}_{{4}} \cdot^{ - } + {\text{ 2OH}}^{ - } + {\text{CO}}_{{2}} $$

Similarly, Fig. 4c shows that H_2_PO_4_^-^ degrades TC rapidly, which may be due to the transformation of SO_4_·^−^ into the more reactive H_2_PO_4_·^−^ as shown in(Eqs. 11 and 12)^48^.

11 $$ {\text{SO}}_{{4}} \cdot^{ - } + {\text{ H}}_{{2}} {\text{PO}}_{{4}}^{ - } \to {\text{ SO}}_{{4}}^{{{2} - }} + {\text{ H}}_{{2}} {\text{PO}}_{{4}} \cdot^{ - } $$

12 $$ {\text{HO}} \cdot \, + {\text{ H}}_{{2}} {\text{PO}}_{{4}}^{ - } \to {\text{ OH}}^{ - } + {\text{ H}}_{{2}} {\text{PO}}_{{4}} \cdot^{ - } $$

### Reusability of ZnFe_2_O_4_ in catalytic reaction

**Figure 5:**
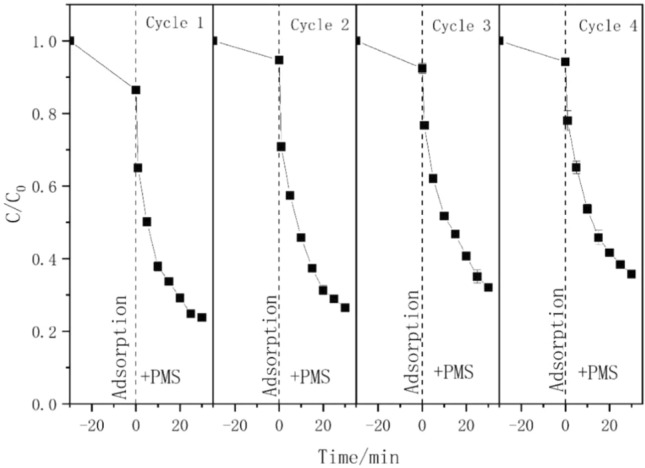
TC degradation in four successive operation catalyzed by ZnFe_2_O_4_. ([Catalyst] = 200 mg L^−1^, [TC] = 20 mg L^−1^, [PMS] = 100 mg L^−1^, pH = 7).

The most crucial factor in practical applications is the capacity of the catalyst to be reused. To investigate the reusability of ZnFe_2_O_4_, four cycling runs were performed under ideal experimental conditions. As shown in Fig. 5, the TC degradation efficiencies reduced from 77 to 66% in 60 min after 4 cycles, indicating the good reusability of the ZnFe_2_O_4_ catalyst. A minor metal ion overflow on the catalyst may have decreased its activity. Furthermore, TC decomposition may have been hampered by intermediate products absorbed by the catalyst^49^.

### Catalytic mechanism

**Figure 6:**
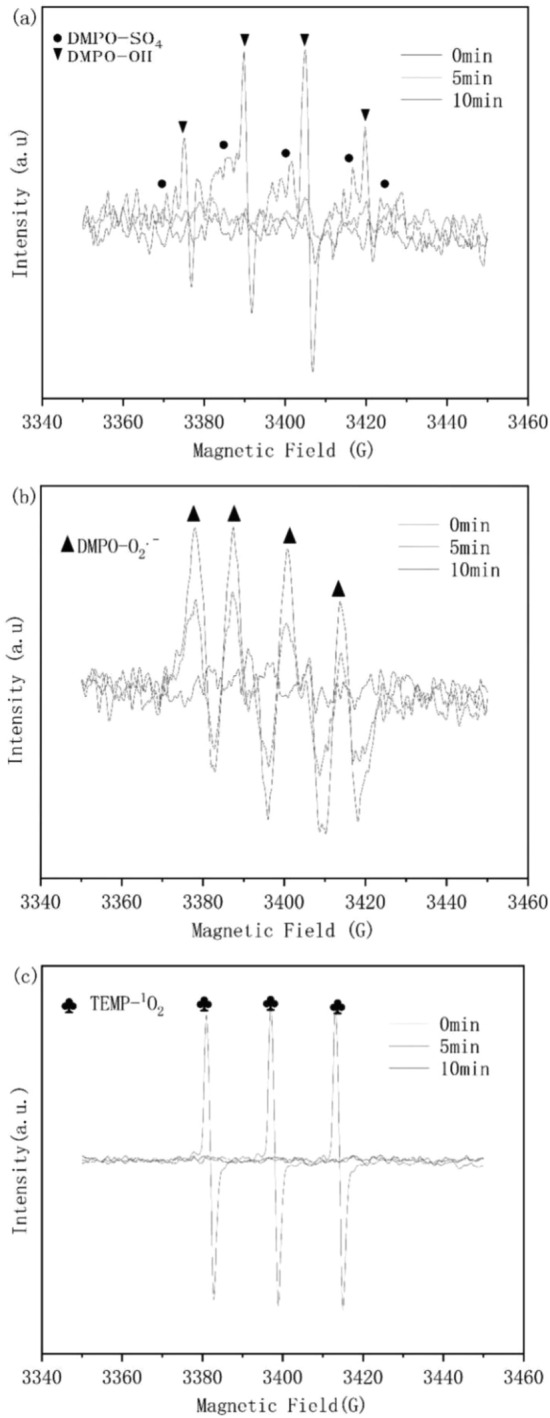
EPR spectroscopy measurements of (**a**) SO_4_·^−^ and HO·, (**b**) O_2_·^−^ and (**c**) ^1^O_2_.

To explore the catalytic mechanism of ZnFe_2_O_4_, ROS involved in the ZnFe_2_O_4_/PMS system were investigated using EPR spectroscopy. DMPO was used to capture SO_4_·^−^, HO·, and O_2_·^−^ using spin trapping, and TEMP was used to detect ^1^O_2_. As shown in Fig. 6a, the DMPO-HO· and DMPO- SO_4_·^−^ adducts showed their characteristic peaks when the time was increased from 0 to 10 min. Moreover, the DMPO-O_2_·^−^ adduct signal in Fig. 6b indicates that O_2_·^−^ may also be involved in TC degradation. Moreover, the TEMP-^1^O_2_ adduct signal was detected at 10 min, implying the presence of ^1^O_2_ in the reaction (Fig. 6c). These findings demonstrated that PMS might be triggered by ZnFe_2_O_4_ producing some active substance that removes TC.

**Figure 7:**
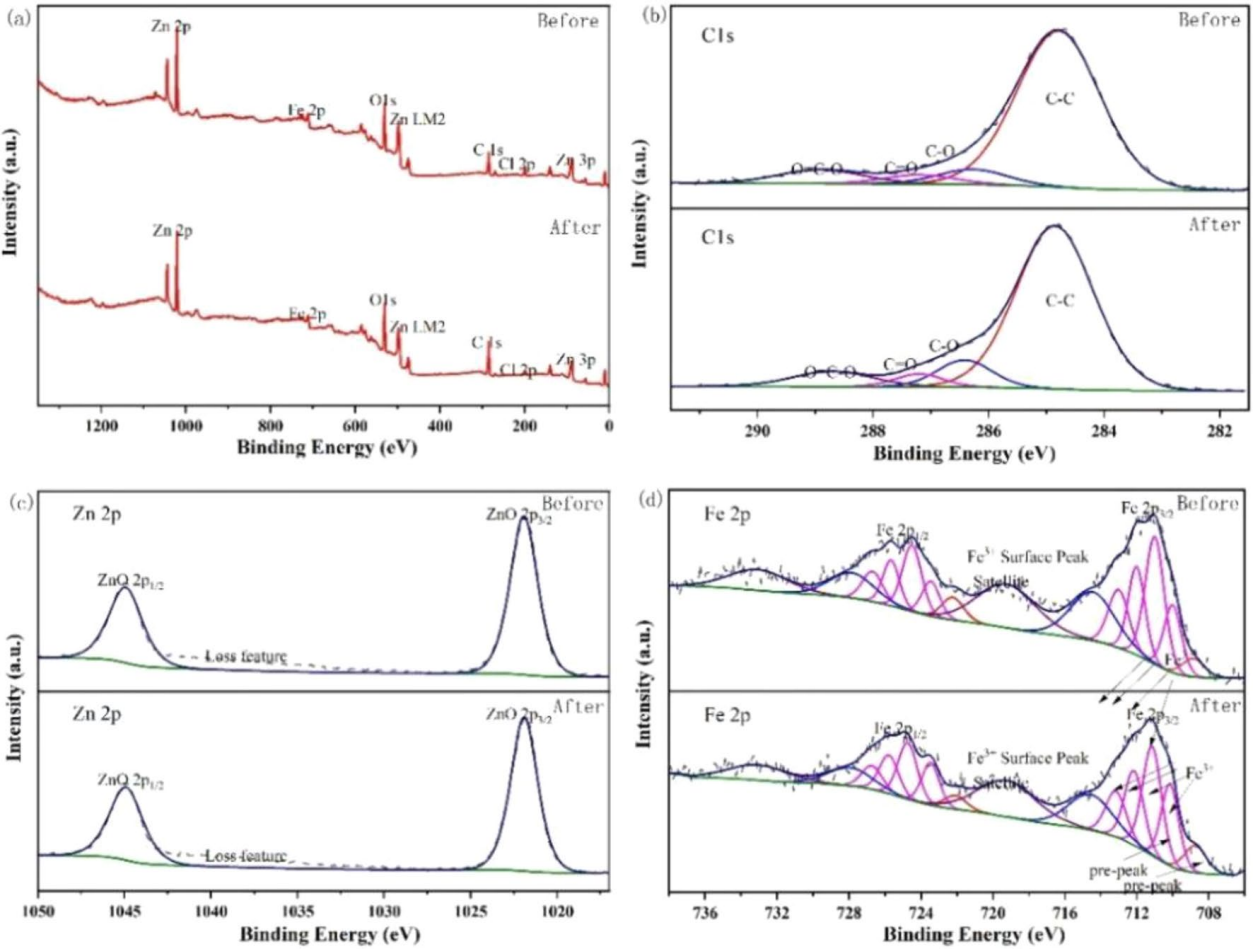
XPS spectra of (**a**) survey, (**b**) C1*s*, (**c**) Zn 2*p*, (**d**) Fe 2*p* for fresh and used ZnFe_2_O_4_ catalyst.

XPS was used to analyse changes in Zn and Fe valence states in untreated and treated ZnFe_2_O_4_ catalysts to further investigate their role in PMS activation. The C 1*s*, O 1*s*, Fe 2*p*, and Zn 2*p* peaks of both new and used catalysts in Fig. 7 indicate the good stability of ZnFe_2_O_4_. The peaks at 284.8, 530.8, 711.5, and 1021.6 eV in Fig. 7a correspond to C 1*s*, O 1*s*, Fe 2*p*, and Zn 2*p*, respectively. Table 2 shows the relative element contents before and after the reaction. The C 1*s* orbital in the samples before and after the reaction has similar components, which can be identified as C–O, C–C, O=C–O, and C=O based on the peak patterns and binding energies of 286.3, 284.8, 288.9, and 287.2 eV, respectively (Fig. 7b and Table 3). In Fig. 7c, the energy difference between the spin–orbit splitting peaks (2*p*3/2 and 2*p*1/2) is approximately 23 eV, and the spectral peak area ratio (2*p*3/2:2*p*1/2) is approximately 2:1. The energy position of Zn 2*p* spectral peaks and the database suggest that 1021.9 eV should correspond to ZnO. Based on the Fe 2*p* spectrum in Fig. 7d, the Fe species in the catalyst should be Fe^3+^ with the lowest peak intensity at 708.9 eV. The peaks at 714.5 and 719.3 eV are surface and satellite peaks of the catalyst, respectively, whereas those at 710.0, 711.0, 712.0, and 713.0 eV correspond to the four typical multiple cleavages of Fe^3+^ with relative contents listed in Table 4. Based on these results, the valence states of Fe and Zn in the catalyst have not changed substantially. However, following the reaction, the carbon content rose, whereas the concentrations of Fe and Zn decreased. Although the catalyst contains only Fe^3+^, its content at a low binding energy increased after the reaction.

**Table 2:** Relative content of the elements in the samples/at.%.

Sample	C	O	Zn	Fe
1	42.96	35.44	13.97	7.63
2	52.09	32.84	9.80	5.27

**Table 3:** Relative content of each chemical bond of C1s/at.%.

Sample	C–C	C–O	C=O	O=C=O
1	82.03	6.52	4.07	7.38
2	79.22	9.26	3.88	7.64

**Table 4:** Relative content of each chemical bond of Fe^3+^/at.%.

Sample	1-Fe^3+^	2-Fe^3+^	3-Fe^3+^	4-Fe^3+^
1	18.96	38.70	23.33	19.01
2	24.48	35.37	24.15	16.00

### Degradation pathways of TC

**Figure 8:**
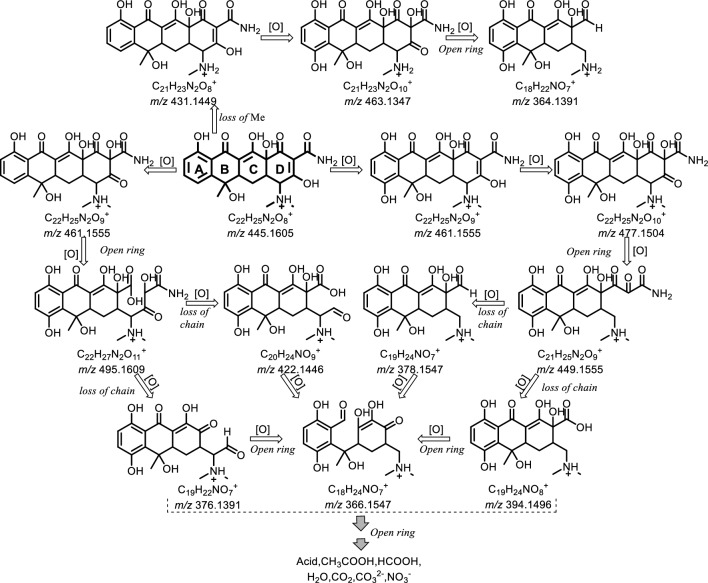
Degradation pathways and proposed mechanism of tetracycline.

To elucidate the TC decomposition process, the main products were qualitatively analysed using the ESI Q Orbitrap HRMS. Figure 8 shows the possible degradation pathways discussed below. The initial compound (TC) has an ion peak at *m/z* 445, corresponding to the proton-ionised form of [M + H]^+^. The first possible reaction pathway involves the formation of the *m/z* 461 product via oxidative hydroxylation of the “A” ring of TC, *m/z* 477 via hydroxylation of the “D” ring, and *m/z* 449 via oxidative “D” ring opening and removing the carbonyl group. Further oxidation could occur when the C–C bonds are broken and side chains are removed to yield *m/z* 378 and 394. In addition, *m/z* 366 could be obtained via the oxidative ring opening of the “B” ring. The second possible reaction pathway involves the formation of product *m/z* 461 via hydroxylation of the “D” ring, product *m/z* 495 via the “D” ring opening, and products *m/z* 376 and 422 via oxidative breaking of the C–C bonds. The product *m/z* 366 was then obtained by oxidising the “B” ring and removing the carboxyl group. The third possible reaction channel involves the TC breakdown via the C–N bond and demethylation of dimethylamine to procure *m/z* 431, the hydroxylation of the “A” ring of TC to obtain *m/z* 463, and the oxidative opening of the “D” ring to procure *m/z* 364. The splitting of the rings to form tiny molecules of acids and amines, as well as H_2_O, CO_2_, ${\text{NO}}_{3}^{ - }$, and ${\text{NH}}_{{4}}^{ + }$, indicate oxidative breakdown and complete degradation of the compounds.

## Conclusions

The co-precipitation method was used to synthesise a highly active ZnFe_2_O_4_ catalyst, which was subsequently used as a PMS activator to degrade TC. The ZnFe_2_O_4_ catalyst was studied using XRD, SEM, and XPS. The effects of the changes in the amount of catalyst, PMS concentration, and initial pH on TC decomposition efficiency were studied under various conditions. Under the optimised reaction conditions, the TC degradation efficiency reached 78%. Inorganic anions (H_2_PO_4_^–^${\text{H}}_{{2}} {\text{PO}}_{{4}}^{-}$, ${\text{CO}}_{{3}}^{{{2}{-}}}$, and Cl^–^) can promote TC degradation to certain extent. In the cyclic experiments, ZnFe_2_O_4_ was found to be catalytically active and stable. In addition, the EPR and XPS results revealed the presence of numerous active substances, including SO_4_·^–^, HO·, O_2_·^–^, and ^1^O_2_, in the TC mineralization process. The potential TC degradation mechanisms of the ZnFe_2_O_4_/PMS system were hypothesised to depend on the products identified by UPLC-MS. These results demonstrated that the prepared ZnFe_2_O_4_ catalyst effectively removed TC and exhibited excellent catalytic performance.

## Data Availability

Data is available under reasonable request to the corresponding author.
